# Variability in respiratory rhythm generation: in vitro and in silico models

**DOI:** 10.1186/1471-2202-15-S1-P12

**Published:** 2014-07-21

**Authors:** Chris Fietkiewicz, Christopher G Wilson

**Affiliations:** 1 Dept. of Elec. Eng. and Comp. Sci., Case Western Reserve University, Cleveland, OH, 44107, USA; 2 Division of Physiology, School of Medicine, Loma Linda University, Loma Linda, CA, 92350, USA

## 

The variability inherent in rhythmic physiological patterns may arise from both stochastic and deterministic sources. Though variability is disruptive when in excess, it may also be functional in evolved systems. Here we focus on the neural control of respiration which is critical for survival in many animals. The sources of respiratory variability, as well as its possible function, are unknown. A fundamental component of respiratory pattern generation is the preBötzinger complex (preBötC) which is part of the ventral respiratory column located in the brainstem. Here we use in vitro and in silico models of the preBötC to study the variability of respiratory rate.

The in vitro model exhibits a bounded range of variability in which the upper and lower limits are functions of the respiratory rate. The in silico model consists of two types of neurons: intrinsically bursting “pacemaker” cells and tonically spiking cells that relay chemosensory and mechanosensory feedback. When fitted to the experimental data, the in silico model shows the existence of both stochastic and deterministic sources for variability in the respiratory rate. The upper and lower limits of variability seen experimentally are reproduced in silico using a range of network connection strengths. Simulations demonstrate that stochastic spiking in sensory relay neurons may be utilized to stabilize changes in variability when the respiratory rate changes due to physiological demand.

